# Postural control adaptations to different visual conditions in dancers and non-dancers with chronic ankle instability

**DOI:** 10.3389/fspor.2025.1553847

**Published:** 2025-04-15

**Authors:** Eun Ji Hong, Jiho Kang, Hyung Gyu Jeon, Sae Yong Lee, Kyeongtak Song

**Affiliations:** ^1^ Department of Kinesiology, Yonsei University, Seoul, Republic of Korea; ^2^ School of Health and Kinesiology, University of Nebraska at Omaha, Omaha, NE, United States; ^3^ Department of Physical Education, Yonsei University, Seoul, Republic of Korea; ^4^ International Olympic Committee Research Centre Korea, Yonsei University, Seoul, Republic of Korea

**Keywords:** balance, postural stability, center of pressure, visual reliance, ankle injury, drop vertical jump

## Abstract

**Purpose:**

Even though dancers have good postural control ability, ankle sprain is the most common injury among dancers, developing into chronic ankle instability (CAI). While dancers rely on visual cues during stage and practice, it is unknown how dancers with CAI perform balance in different visual conditions. This study compares (1) static postural control between eyes open and closed, (2) visual reliance, and (3) dynamic postural control among dancers with CAI, uninjured dancers, non-dancers with CAI, and uninjured non-dancers.

**Methods:**

Ten dancers with CAI, 10 uninjured dancers, 10 non-dancers with CAI, and 10 uninjured non-dancers. Participants performed single-leg standing with eyes open (EO) and eyes closed (EC) conditions. COP area, velocity in mediolateral (ML) and anteroposterior (AP), and resultant velocity were calculated. Visual reliance (% modulation) was calculated using the percent change in COP values between EO and EC conditions. Participants performed a drop vertical jump and maintained balance to assess the dynamic postural stability index. A two-way analysis of variance (group and CAI status) and Bonferroni *post hoc* test were used to compare static balance, visual reliance, and dynamic balance.

**Results:**

A main effect of CAI status was observed in COP area (*p* = 0.014) and COP AP velocity (*p* = 0.013) during static balance in the EO condition. We also observed CAI status main effect in COP area (*p* = 0.014), COP AP velocity (*p* = 0.010), and COP resultant velocity (*p* = 0.034), and a group main effect in COP ML velocity (*p* = 0.034) in EC condition. We found interactions between group and CAI status in the visual reliance of COP resultant velocity (*p* = 0.048), as well as significant group (*p* < 0.001) and CAI status effect (*p* = 0.006). However, there were no significant differences in dynamic postural control (*p* > 0.05).

**Conclusion:**

CAI patients demonstrated postural control deficits in static balance under both eyes open and closed conditions compared to uninjured controls. However, dancers exhibited higher visual reliance than non-dancers, and CAI showed greater visual dependence than uninjured controls. This finding shows dancers use visual information differently, resulting in higher balance abilities.

## 1 Introduction

Dance involves complex movements such as *changement*, *sissonne*, and *pirouette* that exceptional balance and multi-joint coordination of the lower extremity (1, 2). This high-level physical activity requires superior single-leg balance, both static and dynamic conditions, particularly during activities like jumping and landing (3). Consequently, the repetition nature of single-leg postural control movements allows dancers to develop enhanced static and dynamic postural stability compared to non-dancers (4, 5) and athletes in other sports, such as soccer (6, 7). This superior balance performance may be attributed to enhanced neuromuscular control of the ankle, lower thresholds of plantar cutaneous sensitivity, and an improved ankle joint position sense observed in dancers (6, 8). However, despite these advanced abilities, dancers are still prone to ankle injuries, particularly during landings following jumps (9).

Ankle sprains are the most common musculoskeletal injuries among dancers, with a reported prevalence of 35.8% (9). Following a lateral ankle sprain (LAS), dancers frequently experience residual symptoms and recurrent episodes of “giving way” (10), with 75.9% progressing to chronic ankle instability (CAI) (11). CAI not only hinders performance but is also linked to early retirement and long-term complications such as post-traumatic ankle osteoarthritis (12–14). Although dancers undergo rigorous training to recover and maintain performance despite ankle injuries the mechanism underlying recurrent ankle sprains in dancers remains unclear (10). This paradox, where superior balance abilities coexist with a high prevalence of injuries, emphasizes the need for a deeper understanding of balance and sensory processing in dancers with CAI.

Maintaining balance requires both appropriate motor responses and sensory feedback from the visual, vestibular, and somatosensory systems (15, 16). Impaired postural control, resulting from mechanoreceptor disruption in the ligaments and joint capsule following an ankle injury, is a hallmark of CAI (17). These postural control deficits increase the risk of recurrent ankle sprains (18). However, unlike typical individuals with CAI, dancers sustain recurrent ankle injuries despite having superior postural control. For example, dancers with CAI demonstrate superior dynamic balance compared to non-dancers with CAI (19). This may indicate that dancers may have different mechanisms of ankle sprains compared to the general CAI population. Recently, altered sensory organization strategies during postural control have been proposed as a risk factor for postural instability. Specifically, individuals with CAI rely more on visual information during single-leg stance due to reduced somatosensory feedback from the foot-ankle complex, compared to healthy controls (20). However, most previous studies have compared static balance ability between uninjured dancers and non-dancers (21), with limited research on postural control ability and visual reliance in dancers with CAI vs. non-dancers with CAI. Thus, to prevent recurrent ankle sprains in dancers, it is important to comprehensively examine both postural control and visual reliance across dancer and non-dancer groups with and without CAI.

Therefore, the purpose of this study was to compare (1) single limb static postural control, (2) visual reliance during static balance, and (3) dynamic postural control during jump landing among the dancers with CAI, uninjured dancers, non-dancers with CAI, and uninjured non-dancers. We hypothesized that, compared to non-dancers with CAI and uninjured non-dancers, dancers with CAI and uninjured dancers would demonstrate (1) greater static balance ability in the eyes open condition, (2) higher visual reliance, and (3) better dynamic balance ability.

## 2 Methods

### 2.1 Participants

A*priori* estimated sample sizes for *α* level = 0.05, a power = 0.80, and a partial eta squared = 0.26 were calculated based on time-to-boundary balance variable data from previous research, which investigated individuals with CAI during single-leg balance (22). Using G*Power software (ver. 3.1.9.2, Kiel, Germany), 40 participants were recruited. All female participants aged between 20 and 35 were voluntarily recruited for this study. The participants were divided into 4 groups based on their ankle conditions, dance experience, and Tegner activity level: dancers with CAI (*n* = 10), uninjured dancers (*n* = 10), non-dancers with CAI (*n* = 10), and uninjured non-dancers (*n* = 10), as shown in Table 1. The inclusion criteria for participants in the CAI groups (both dancers and non-dancers) followed the guidelines of the International Ankle Consortium (23). These criteria were as follows: (a) a history of at least one lateral ankle sprain; (b) at least two episodes of “giving way” in the past 6 months; (c) a Foot and Ankle Ability Measure (FAAM)-Activities of Daily Living (ADL) subscale score of less than 90%; (d) a FAAM-Sports subscale score of less than 80%; (e) an Identification of Functional Ankle Instability (IdFAI) subscale score of 11 or higher. The participants in the control groups met the following criteria: (a) no history of lateral ankle sprain; (b) a 100% score on both the FAAM-ADL and Sports; (c) an IdFAI subscale score of less than 11. Exclusion criteria for all participants included: (a) any acute lower-extremity musculoskeletal injuries occurring within 3 months prior to study enrollment; and (b) a history of lower-extremity surgery and/or fracture that could affect balance.

**Table 1:** Demographic characteristics mean (SD) for each group.

Variables	Dancers with CAI (*n* = 10)	Uninjured dancers (*n* = 10)	Non-dancers with CAI (*n* = 10)	Uninjured non-dancers (*n* = 10)	*p*
Age (year)	22.10 (2.81)	20.90 (1.66)	23.80 (4.05)	22.20 (2.49)	0.183
Height (cm)	166.63 (4.01)	164.93 (4.87)	165.40 (7.13)	161.00 (5.62)	0.141
Weight (kg)	52.96 (5.24)	53.93 (4.56)	61.10 (3.84)	53.18 (5.02)	<0.001
IdFAI (%)	18.70 (4.17)	0.0 (0.0)	21.20 (4.92)	0.0 (0.0)	<0.001
FAAM-ADL (%)	81.79 (8.53)	100.0 (0.0)	77.26 (11.0)	100.0 (0.0)	<0.001
FAMM-sports (%)	67.86 (11.66)	100.0 (0.0)	71.43 (10.48)	100.00 (0.0)	<0.001
Tegner	6.20 (0.79)	7.20 (1.32)	5.00 (1.05)	5.60 (0.52)	<0.001
Dance experience (year)	9.70 (2.21)	10.10 (2.69)	0.0 (0.0)	0.0 (0.0)	0.150

ADL, activities of daily living; CAI, chronic ankle instability; FAAM, foot and ankle ability measure; IdFAI, identification of functional ankle instability.

To qualify as dancers, participants were required to have at least 7 years of ballet or modern dance training at a professional dance academy registered to establish and operate a dance-educational institute, or at an arts middle/high school (24). They must be current students or graduates of a 4-year university, actively engaged as professional dancers. Non-dancers had no prior formal dance experience. All participants provided written informed consent prior to the start of the experiment. This study was approved by the Institutional Review Board of Yonsei University (IRB No. 7001988-202209-HR-1691-02 & 7001988-202207-HR-1631-02).

### 2.2 Experimental procedure

The height, weight, age, and leg length of the participants were recorded, followed by a 5-minute warm-up involving walking and jogging prior to data collection. Dancers and non-dancers with CAI performed the tests using their injured limbs, while dancers and non-dancers without CAI used their dominant limbs. The dominant limb was defined as the leg preferred for kicking a ball (25).

Single-leg balance was performed to assess static postural stability and was evaluated using a force plate (Accusway Plus, AMTI, Watertown, MA) with a sampling rate of 50 Hz (26). Participants performed a barefoot single-limb stance on the force plate under both eyes-open and eyes-closed conditions for 10 s. According to previous studies, a 10-second single-leg balance test has been sufficient to detect balance differences between individuals CAI and uninjured controls (27). All participants were first assessed for single-leg balance under the eyes-open condition, followed by the eyes-closed condition. They were instructed to maintain balance while focusing on a visual target positioned 1 m ahead for the eyes-open condition. Participants were instructed to keep their hands on their waist, look straight ahead, and hold the non-testing leg at approximately 45° of knee flexion and 30° of hip flexion (28). During the eyes-closed trials, participants maintained the same posture as in the eyes-open trials, but with their eyes closed. All participants completed at least three trials in each condition to familiarize themselves with the task. Errors were determined based on the following conditions: (1) touching the ground with the non-testing leg, (2) lifting or moving the testing leg away from the force plate, or (3) being unable to maintain balance for the 10-second duration (29). Trials in which participants lost balance or failed to complete the task were excluded from the data, and the trial was repeated. Participants continued the trials unless they experienced pain or expressed a desire to stop. All participants completed three successful trials for each condition, which were recorded and analyzed.

The drop vertical jump (DVJ) landing task was used to assess dynamic postural control, as it is more challenging than static postural control and requires coordinated movement of the entire lower extremity upon landing (30). According to previous studies, individuals with CAI demonstrated dynamic postural stability deficits during anterior unilateral jump landing tasks compared to healthy controls (31, 32). The box height and its distance from the force plate were set to 50% of each participant's height (33). Participants stood on a box with involved legs, dropped off the box, and landed on both feet, with each foot landing separately on a force plate (34). After ground contact, the participant immediately performed a maximal vertical jump and landed again on the force plates using the testing leg (34). After landing, participants were instructed to immediately keep the hip and knee joints of the non-testing leg bent at 90° and hold for at least 1 s. Errors were identified under the following conditions (30): (1) stepping down with the non-testing leg or failing to land entirely on the force plate with the testing leg, (2) hopping or shifting the testing leg on the force plate, or (3) losing balance for 1 s. Each participant completed three successful trials.

### 2.3 Data reduction

Center of pressure (COP) data were calculated using Balance Clinic Software (ver. 2.02.01; AMTI, Watertown, MA, USA) and filtered with a fourth-order, zero-lag, low-pass filter with a cut-off frequency of 5 Hz (35). In this study, four variables were analyzed (35): (1) the COP area of the 95% confidence ellipse, (2) mean COP velocity in AP, (3) mean COP velocity in ML, and (4) COP resultant velocity. The 95% COP area ellipse reflects the magnitude of postural sway, while COP velocity indicates the regulatory activity of the postural control system (36). Both outcome measures have revealed significant differences between stable and unstable ankles (37). The greater COP area, as well as the mean COP velocity in AP, ML, and COP resultant velocity, indicates worse postural control ability.

To quantify the decline in postural control from eyes open to eyes closed conditions (visual reliance), we computed the outcome variable by averaging the trials for each COP parameter. The visual reliance (% modulation) was calculated using the formula: (Eyes open COP–Eyes closed COP)/Eyes open COP × 100 (20). Briefly, the change in the percentage score indicates the extent of the decline in static postural control that results from the removal of visual input, thereby reflecting the level of reliance on visual; higher values suggest a greater reliance on visual cues.

Dynamic postural was defined as when the vertical ground reaction force (vGRF) exceeded 10 N after second landing on the testing leg. vGRF were normalized by the participant's body weight. All data collected using a force plate (ORG-6 AMTI, Watertown, MA, USA) were collected at a sampling rate of 2,000 Hz and filtered using the 4th-order low-pass Butterworth filter at 5 Hz. Dynamic postural stability index (DPSI) scores were assessed for a 1-second and calculated with an Equation to obtain indices in three principal directions (38). We analyzed a short period after landing because postural control ability immediately after landing is crucial, and a shorter sampling interval has been found to be more sensitive for assessing dynamic postural stability than a longer interval (39). The mediolateral stability index [MLSI], anteroposterior stability index [APSI], and vertical stability index [VSI] correspond with the frontal (X), sagittal (Y), and transverse (Z) axes of the force plate. These indices measure the standard deviation of fluctuations around a zero point, which is then divided by the number of data points in a trial, with higher scores indicating greater variability.

Equation postural stability index in three directions.

$$\mathrm{APSI}=\sqrt{\left[\sum {\left(O-Y\right)}^{2}/\mathrm{number}\;\mathrm{of}\;\mathrm{data}\;\mathrm{points}\right]}$$

$$\mathrm{MLSI}=\sqrt{\left[\sum {\left(O-X\right)}^{2}/\mathrm{number}\;\mathrm{of}\;\mathrm{data}\;\mathrm{points}\right]}$$

$$\mathrm{VSI}=\sqrt{\left[\sum {\left(\mathrm{body}\;\;\mathrm{weight}-Z\right)}^{2}/\mathrm{number}\;\mathrm{of}\;\mathrm{data}\;\mathrm{points}\right]}$$

### 2.4 Statistical analysis

One-way analysis of variance (ANOVA) was conducted to evaluate differences in demographics among the groups, with significant findings identified using the Bonferroni *post hoc* test. Additionally, independent *t*-tests were performed to compare the duration of dance experience within the dance groups (dancers with CAI and uninjured dancers).

Univariate two-way analyses of covariance (ANCOVA) were conducted to identify differences between groups and CAI status for the dependent variables. The independent variables were group (dancers and non-dancers) and CAI status (CAI and uninjured controls). The dependent variables were COP area, COP ML and AP velocity, COP resultant velocity during static balance with eyes open and closed conditions, visual reliance, and dynamic balance. Since there were differences in weight among groups (*p* < 0.001), weight was included as a covariate due to its potential effect on postural control (40). After adjusting for weight as a covariate, the statistical significance of the primary effect remained. When significant interactions between the group and CAI status were observed, Bonferroni-corrected *post hoc* pairwise comparisons were conducted to determine the location of significance. Partial eta squared (*η*^2^_p_) was calculated to estimate effect sizes, classified as small (0.01–0.06), moderate (0.06–0.14), and large (≥0.14). Statistical significance was set at *p* < 0.050, and all analyses were conducted using SPSS 28.0 (IBM Corp, Armonk, NY, USA).

## 3 Results

Table 1 presents the demographic data and questionnaire scores of this study. A significant difference in weight was observed (*p* < 0.001), with non-dancers with CAI exhibiting higher weight compared to dancers with CAI (*p* = 0.003), uninjured dancers (*p* = 0.010), and uninjured non-dancers (*p* = 0.004). Due to the recruitment criteria, individuals with CAI showed a significantly higher IdFAI score, and lower FAAM-ADL and FAAM-Sports scores compared to uninjured controls (*p* < 0.001), regardless of dance groups. Additionally, uninjured dancers demonstrated a higher Tegner activity level than the non-dancers with CAI (*p* < 0.001), and uninjured non-dancers (*p* = 0.004). Lastly, there was no significant difference in the duration of dance experience between dancers with CAI and uninjured dancers (*p* = 0.150).

### 3.1 Static postural control

#### 3.1.1 Single leg balance with eyes open

Table 2 represents the single-leg postural ability under the eyes-open condition after adjusting for weight as a covariate. No significant interactions between the group and CAI status or main effects of the group were observed across all variables (*p* > 0.05). However, a significant main effect of CAI status was found. Specifically, the CAI group exhibited a larger COP area (*p* = 0.014) and higher COP AP velocity (*p* = 0.013) than the uninjured healthy controls, indicating that individuals with CAI demonstrated poorer eyes open postural control regardless of dance experience.

**Table 2:** Mean (SD), *F* scores, *p* values, and effect sizes of postural control variables in the single leg balance with eyes open.

Variables	CAI status	Dance group		Statistical values								
		Dancers	Non-dancers	Group X CAI interaction			Group main effect			CAI status main effect		
				*F*_1, 36_	*p*	*η* ^2^ *_p_*	*F*_1, 36_	*p*	*η* ^2^ *_p_*	*F*_1, 36_	*p*	*η* ^2^ *_p_*
COP area (cm^2^)^a^	CAI	5.27 (1.38)	7.80 (4.09)	2.417	0.129	0.065	3.413	0.073	0.089	6.661	0.014	0.160
	Uninjured controls	4.44 (1.49)	4.67 (1.35)									
COP ML velocity (cm/s)	CAI	0.88 (0.20)	1.02 (0.19)	2.085	0.158	0.056	2.480	0.124	0.066	1.924	0.174	0.052
	Uninjured controls	0.88 (0.13)	0.90 (0.22)									
COP AP velocity (cm/s)^a^	CAI	0.84 (0.19)	0.97 (0.34)	1.659	0.206	0.045	1.785	0.190	0.049	6.856	0.013	0.164
	Uninjured controls	0.74 (0.12)	0.74 (0.15)									
COP resultant velocity (cm/s)	CAI	3.83 (0.76)	4.55 (1.05)	2.388	0.131	0.064	3.774	0.060	0.097	2.819	0.102	0.075
	Uninjured controls	3.78 (0.46)	3.90 (0.88)									

Weight was not found to be a significant covariate for any of the balance variables with eyes open (*p* > 0.05). AP, anteroposterior; CAI, chronic ankle instability; COP, center of pressure; ML, mediolateral; SD, standard deviation.

^a^ Indicates that the CAI group exhibits greater (worse postural control) values than the uninjured control group.

#### 3.1.2 Single leg balance with closed eyes

Table 3 shows the single-leg postural ability under the eyes-closed condition after accounting for weight as a covariate. No significant interactions between the group and CAI status were observed during single-leg balance with eyes closed (*p* > 0.05). However, a significant main effect of the group was found for COP ML velocity, with dancers exhibiting higher values than non-dancers (*p* = 0.044). Additionally, a significant main effect of CAI status was observed. Specifically, individuals with CAI demonstrated higher values (indicating poorer postural control) than uninjured healthy controls in COP area (*p* = 0.014), COP AP velocity (*p* = 0.010), and COP resultant velocity (*p* = 0.034).

**Table 3:** Mean (SD), *F* scores, *p* values, and effect sizes of postural control variables in the single leg balance with eyes closed.

Variables	CAI status	Dance group		Statistical values								
		Dancers	Non-dancers	Group X CAI interaction			Group main effect			CAI status main effect		
				*F*_1, 36_	*p*	*η* ^2^ *_p_*	*F*_1, 36_	*p*	*η* ^2^ *_p_*	*F*_1, 36_	*p*	*η* ^2^ *_p_*
COP area (cm^2^)^a^	CAI	22.02 (7.44)	23.99 (6.16)	2.684	0.110	0.071	0.645	0.427	0.018	6.647	0.014	0.160
	Uninjured controls	20.14 (6.76)	14.70 (4.32)									
COP ML velocity (cm/s)^b^	CAI	2.08 (0.33)	1.94 (0.41)	0.164	0.688	0.005	4.352,	0.044	0.111	2.232	0.144	0.060
	Uninjured controls	1.96 (0.33)	1.66 (0.29)									
COP AP velocity (cm/s)^a^	CAI	1.91 (0.40)	1.91 (0.53)	3.219	0.081	0.084	1.556	0.221	0.043	7.392	0.010	0.174
	Uninjured controls	1.79 (0.31)	1.37 (0.30)									
COP resultant velocity (cm/s)^a^	CAI	8.94 (1.29)	8.94 (1.92)	2.340	0.135	0.063	2.343	0.135	0.063	4.843	0.034	0.122
	Uninjured controls	8.63 (1.10)	7.08 (1.32)									

Weight was not found to be a significant covariate for any of the balance variables with eyes closed (*p* > 0.05). AP, anteroposterior; CAI, chronic ankle instability; COP, center of pressure; ML, mediolateral; SD, standard deviation.

^a^ Indicates that the CAI group exhibits greater (worse postural control) values than the uninjured control group.

^b^ Indicates that the dance group exhibits greater (worse postural control) values than the non-dancer group.

### 3.2 Visual reliance during static postural control

The results comparing visual reliance during postural control across groups and CAI status, adjusting for weight as a covariate, are shown in Table 4. A significant interaction between group and CAI status was found for COP resultant velocity (*p* = 0.048). Dancers with CAI (*p* < 0.001), uninjured dancers (*p* < 0.001), and non-dancers with CAI (*p* = 0.016) exhibited worse postural control than uninjured non-dancers. Additionally, a significant main effect of group was observed for COP area (*p* = 0.021), COP ML velocity (*p* = 0.008), and COP AP velocity (*p* = 0.009), with dancers exhibiting greater visual reliance than non-dancers.

**Table 4:** Mean (SD), *F* scores, *p* values, and effect sizes of visual reliance in the single leg balance.

Variables	CAI status	Dance group		Statistical values								
		Dancers	Non-dancers	Group X CAI interaction			Group main effect			CAI status main effect		
				*F*_1, 36_	*p*	*η* ^2^ *_p_*	*F*_1, 36_	*p*	*η* ^2^ *_p_*	*F*_1, 36_	*p*	*η* ^2^ *_p_*
COP area (%)	CAI	−333.09 (158.21)	−242.90 (129.94)	0.349	0.558	0.010	5.831	0.021	0.143	0.114	0.737	0.003
	Uninjured controls	−379.72 (169.14)	−230.21 (103.13)									
COP ML velocity (%)^a^	CAI	−152.68 (70.29)	−94.71 (40.88)	1.047	0.313	0.029	7.940	0.008	0.185	0.184	0.671	0.005
	Uninjured controls	−127.43 (44.71)	−94.69 (47.95)									
COP AP velocity (%)^a^	CAI	−139.80 (64.45)	−106.31 (45.24)	0.913	0.346	0.025	7.537	0.009	0.177	0.210	0.650	0.006
	Uninjured controls	−147.35 (26.22)	−89.89 (34.42)									
COP resultant velocity (%)^a^^,^^b^	CAI	−139.90 (51.88)	−100.43 (39.15)	4.187	0.048	0.107	30.344	<0.001	0.464	8.520	0.006	0.196
	Uninjured controls	−129.97 (26.87)	−23.51 (39.00)									

Weight was not found to be a significant covariate for any of the visual reliance measures (*p* > 0.05). AP, anteroposterior; CAI, chronic ankle instability; COP, center of pressure; ML, mediolateral; SD, standard deviation.

^a^ Indicates that the CAI group exhibits greater (worse postural control) values than the uninjured control group.

^b^ Indicates that the dance group exhibits greater (worse postural control) values than the non-dancer group.

### 3.3 Dynamic postural control

No significant interactions and main effects were observed for dynamic postural control variables (*p* > 0.05), with weight adjusted as a covariate. Table 5 presents the APSI, MLSI, and VSI scores for dynamic postural control across groups and CAI status.

**Table 5:** Mean (SD), *F* scores, *p* values, and effect sizes of postural control variables in the dynamic balance.

Variables	CAI status	Dance group		Statistical values								
		Dancers	Non-dancers	Group X CAI interaction			Group main effect			CAI status main effect		
				*F*_1, 36_	*p*	*η* ^2^ *_p_*	*F*_1, 36_	*p*	*η* ^2^ *_p_*	*F*_1, 36_	*p*	*η* ^2^ *_p_*
MLSI	CAI	0.05 (0.01)	0.04 (0.02)	0.023	0.881	0.001	3.047	0.090	0.080	0.000	0.988	0.000
	Uninjured controls	0.05 (0.01)	0.04 (0.02)									
APSI	CAI	0.05 (0.02)	0.06 (0.01)	1.248	0.272	0.034	0.425	0.519	0.012	1.439	0.238	0.039
	Uninjured controls	0.05 (0.01)	0.04 (0.02)									
VSI	CAI	2.24 (0.21)	2.15 (0.06)	3.053	0.089	0.080	4.327	0.045	0.110	0.248	0.622	0.007
	Uninjured controls	2.16 (0.04)	2.14 (0.04)									

Weight was not found to be a significant covariate for any of the dynamic balance variables (*p* > 0.05). CAI, chronic ankle instability; MLSI, medial/lateral stability index; APSI, anterior/posterior stability index; VSI, vertical stability index.

## 4 Discussion

To our knowledge, we are the first to present static and dynamic balance abilities, as well as visual reliance, in dancers with CAI relative to uninjured dancers, non-dancers with CAI, and uninjured non-dancers. The primary finding of this study indicates that individuals with CAI exhibited worse postural control abilities with their eyes open during the static postural control compared to the uninjured individuals, regardless of dance experience. Second, in the eyes-closed condition, static postural control was poorer in CAI individuals than in the uninjured individuals. Additionally, dancers exhibited reduced postural control with eyes closed than non-dancers. Third, dancers relied more on vision than uninjured non-dancers, regardless of CAI status.

Our results align with previous research (20, 41–43), which indicates that the CAI group exhibits deficits in postural control compared to uninjured groups during single-leg static balance with their eyes open. This may be attributed to reduced proprioception around the ankle joint, decreased muscle strength, or neuromuscular control deficits. This study expected that dancers with CAI would have excellent balance despite experiencing injuries; however, no interaction was observed. Notably, however, in numerical terms, dancers with CAI showed similar balance abilities to the uninjured groups (uninjured dancers and uninjured non-dancers). This suggests that dancers may develop compensatory mechanisms through extensive technical training, thereby ankle strength, proprioceptive acuity, and visual feedback. For example, dancers undergo “somatic training” that enhances sensory awareness (including proprioception) and refines their biomechanical efficiency—even when injured (44). Dancers also tend to develop superior balance through calf muscle activation, particularly in complex movements (i.e., *pirouettes*) performed on a raised heel (i.e., *demi-pointe* and *en pointe*) (45). Frequent practice and training can therefore promote ankle stabilization and overall balance, potentially enabling dancers to maintain sufficient postural control despite ankle instability. Further research should incorporate more detailed examinations of muscle strength, activation, and co-activation at the ankle joint in dancers with CAI, to better understand how these compensatory mechanisms are established and maintained.

Both dancers and non-dancers with CAI in this study demonstrated diminished static postural control compared to the uninjured group under the eyes-closed condition, suggesting that postural control impairments in CAI groups become more pronounced when visual feedback is removed (27, 28). This finding aligns with previous research indicating that sensorimotor control deficits are more evident in the absence of visual cues, as individuals with compromised ankle stability must then rely on potentially suboptimal joint mechanoreceptors and neuromuscular feedback (27). Moreover, the pronounced negative shifts from eyes open to eyes closed in CAI groups reinforce the critical role of visual input in stabilizing single-leg stance (28). A meta-analysis further demonstrated that individuals with CAI rely more heavily on visual information during single-limb stances compared to uninjured controls (20). Our % modulation results show that uninjured dancers exhibited a modulation of −147.35% compared to uninjured non-dancers (−89.89%). Additionally, dancers with CAI demonstrated a modulation of −139.90% relative to uninjured non-dancers (−85.50%). Overall, altered sensory organization strategies and reduced ability to reweight among different sensory inputs appear to exacerbate postural control deficits when vision is compromised, potentially increasing the risk of recurrent ankle sprains (46).

Many previous studies have reported that uninjured dancers exhibit poorer balance with their eyes closed compared to uninjured non-dancers (45, 47, 48); however, there is a lack of research utilizing a visual dependence formula for dancers. Interestingly, this study reported that dance groups, regardless of an ankle sprain, appear to rely more heavily on visual information. Dancers are specialized in utilizing visual input through mirrors to maintain body alignment and proper posture (45, 48–51), and in increasing visual reliance to prevent collisions or mistakes in their surrounding environment (52). Additionally, dancers rely on visual points to maintain balance control and spatial orientation, which enables high performance in consecutive rotations (i.e., *Fouettés*) (53), and visual fixation allows them to greater advantage of visual information (54). These results indicate that dancers get a lot of visual information when their eyes are open, and visual restriction activities may facilitate the somatosensory information. Conversely, a previous study suggests that the presence or absence of a mirror does not change the dancer's postural control (55). This finding indicates that visual dependence may be increased by other dance-specific characteristics of the environment (e.g., learning movements by watching a teacher or observing a peer). However, the characteristics of dancers who have trained in front of mirrors for an extended period may differ, future study needed to make it necessary to examine postural control both with and without mirrors for considering the duration of training.

In general, CAI patients exhibit impaired dynamic postural control in the AP, ML, and vertical directions along with worse DPSI scores compared to non-dancer controls (38). In this study, while the dancer group showed higher values indicating better balance than non-dancer group, no significant differences were found between groups. Wikstrom et al. (39) recommended selecting the shortest sampling interval that reflects functional sports activities. Nevertheless, the lack of statistical differences may be attributed to the small sample size. However, this study is meaningful as it identifies the characteristics of static balance ability and visual dependency in dancers with CAI. Therefore, future studies should increase sample size, to more clearly distinguish the characteristics of dancers and non-dancers.

There were several limitations. Tegner activity levels were not controlled among the groups, and uninjured dancers in this study were higher than non-dancers with CAI and uninjured non-dancers. To identify the characteristics of dancers, we recruited pre-professional dancers who have been dancing for at least 7 years and are currently active, so there may be differences in activity level compared to the general population. Not only dancers (24) but also gymnasts tend to be active level for high performance. Thus, we cannot rule out the possibility of the activity level of uninjured dancers. Additionally, previous studies have identified the inability to match dominant and non-dominant limbs between the CAI and uninjured control groups as a limitation (56). Similarly, although differences exist between dancers and non-dancers (57), this study did not control these differences. Despite these limitations, this study is significant as it contributes to understanding the characteristics of dancers by comparing their visual dependence based on CAI status to that of non-dancers. This provides a meaningful foundation for proposing new injury prevention strategies tailored to dancers. Finally, to evaluate visual reliance during static balance, a fixed order was used, with the eyes-open condition always preceding the eyes-closed condition. Although this design might have introduced potential order effects or learning bias it was necessary to ensure consistency in comparing postural control across visual conditions. To minimize potential learning effects, all participants completed at least three practice trials under each visual condition and were instructed to utilize their natural single-leg balance strategy. Nevertheless, future research should investigate whether order effects significantly impact visual reliance during single-leg balance.

The clinical implication is that while CAI is known to be associated with balance deficits and increased reliance on visual input, this study is the first to identify that dancers, regardless of CAI, exhibit greater visual dependency. Hutt et al. (48) reported that elite ballet dancers showed a significant increase in dynamic balance after a 4-week intervention with eyes closed compared to the eyes-open group that completed the same exercise. These findings emphasize the importance of studying protocols aimed at reducing visual dependency by maximizing somatosensory function in environments with limited visual input. Cumulatively, reducing reliance on visual information is essential not only for individuals with CAI but also for uninjured dancers. Additionally, dancers are frequently exposed to an environment on stage that, due to the bright lights, is similar to having limited visual input, contributing to high injury rates during activities involving visual limitations, such as rehearsals (58). Dancers may face challenges in changing environments. Therefore, future training programs should consider using stroboscopic glasses to enhance balance under visually confusing conditions.

## 5 Conclusion

Based on this study, regardless of dance experience, individuals with CAI exhibited reduced static postural control ability compared to the uninjured group. However, dancers demonstrated higher visual dependence, and CAI patients showed greater visual reliance than the uninjured controls. A dancer's progression to CAI may be associated with training characteristics such as visual fixation and increased visual utilization. These findings suggest that somatosensory and visual dependency should be assessed separately when evaluating postural control.

## Data Availability

The dataset is available by request to the corresponding authors, subject to institutional and ethical guidelines. Requests to access these datasets should be directed to ktsong@yonsei.ac.kr or sylee1@yonsei.ac.kr.
